# Smoking Status Among Patients With Newly Diagnosed Lung Cancer in Taiwan

**DOI:** 10.1097/jnr.0000000000000293

**Published:** 2019-07-16

**Authors:** Chia-Chen YANG, Chien-Ying LIU, Kwua-Yun WANG, Fur-Hsing WEN, Yu-Chin LEE, Mei-Ling CHEN

**Affiliations:** 1 PhD, RN, Lecturer, School of Nursing, National Defense Medical Center; 2 MD, Associate Professor of Lung Tumor and Endoscopy, Department of Thoracic Medicine, Linkou Chang Gung Memorial Hospital; 3 PhD, RN, FAAN, Dean, Taipei City Chao-Ju Seniors’ Home, and Jointly Appointed Professor, School of Nursing, National Defense Medical Center; 4 PhD, Professor, Department of International Business, School of Business, Soochow University; 5 MD, Associate Professor, Sijhih Cathay General Hospital; 6 PhD, RN, Professor, School of Nursing, College of Medicine, Chang Gung University, and Department of Nursing, Chang Gung University of Science and Technology, and Researcher, Division of Medical Oncology, Department of Internal Medicine, Linkou Chang Gung Memorial Hospital.

**Keywords:** continued smoking, exercise, lung cancer, self-efficacy, smoking cessation

## Abstract

**Background:**

Continued smoking after receiving a diagnosis of cancer seriously affects disease prognosis and survival. The prevalence and risk factors of continued smoking among patients with newly diagnosed lung cancer are unknown in Taiwan.

**Purpose:**

The aims of this study were to assess the smoking status of patients with newly diagnosed lung cancer and to identify the characteristics that are associated with different smoking statuses.

**Methods:**

Baseline data of a longitudinal study on smoking behaviors after lung cancer diagnosis were analyzed in this study. Patients were consecutively recruited from three medical centers in northern Taiwan. A structured questionnaire and medical chart reviews were used to collect data. Multinomial logistic regression analysis was used to examine the factors associated with continuing to smoke after being diagnosed with lung cancer.

**Results:**

Among the 406 patients with newly diagnosed lung cancer who were recruited, 47.0% were never-smokers and 53.0% were ever-smokers. Among the second group, 38% were former smokers, 18% were recent quitters, and 44% were current smokers. Compared with former smokers, current smokers were more likely to be younger (*OR* = 1.05), to not exercise regularly (*OR* = 2.74), to currently live with smokers (*OR* = 2.48), and to have lower self-efficacy for refusing to smoke (*OR* = 0.95). Compared with recent quitters, current smokers were more likely to have lower self-efficacy for refusing to smoke.

**Conclusions/Implications for Practice:**

A significant proportion of ever-smoker lung cancer patients in Taiwan will continue to smoke after receiving their diagnosis. Variables known to modify the risk factors associated with continued smoking such as regular exercise and better refusal self-efficacy should be considered and incorporated into future smoking cessation programs for patients with lung cancer.

## Introduction

Lung cancer, one of the most common cancers worldwide, has the highest mortality rate of all types of cancer (Health Promotion Administration, Ministry of Health and Welfare, Executive Yuan, Taiwan, ROC, 2016b; Siegel, Miller, & Jemal, 2016). Smoking is the primary risk factor for lung cancer (Khuder, 2001). In 1985, smoking was estimated to have contributed to 85%–90% of lung cancer cases in Western countries (Islami, Torre, & Jemal, 2015). However, 25% of patients with lung cancer worldwide have never smoked (Parkin, Bray, Ferlay, & Pisani, 2005), and this proportion appears to be increasing over time (Boffetta, Järvholm, Brennan, & Nyrén, 2001), especially among women (Quoix, 2007). It is estimated that men have been estimated to smoke nearly five times as much as women worldwide, but this ratio varies dramatically across countries (Guindon & Boisclair, 2003). Asian women have a lower rate of smoking than their European and American counterparts (Koo & Ho, 1990). For example, the ratio of male-to-female smokers in Taiwan is 7:1 (Health Promotion Administration, Ministry of Health and Welfare, Executive Yuan, Taiwan, ROC, 2016a), and one study found a history of smoking in only 7% of Taiwanese women with lung cancer (Li, Shieh, & Chen, 2011). Lung cancer in never-smokers, particularly those from Asia, has been suggested to be distinct from that in smokers, with unique clinical features and mortality (Couraud, Zalcman, Milleron, Morin, & Souquet, 2012; Yano et al., 2008).

About half of smokers have been shown to quit smoking when diagnosed with lung cancer (Walker, Larsen, Zona, Govindan, & Fisher, 2004; Walker et al., 2006), but 37%–63.9% of patients with lung cancer continue to smoke after diagnosis (Park et al., 2012; Tseng, Lin, Moody-Thomas, Martin, & Chen, 2012). Continued smoking after lung cancer diagnosis may increase the risk of developing a second, primary smoking-related cancer (Parsons, Daley, Begh, & Aveyard, 2010); poor quality of life (J. Chen et al., 2012); lower performance status (Baser et al., 2006); and shorter survival time (Kovács, Barsai, & Szilasi, 2012; Parsons et al., 2010).

The known adverse effects of postdiagnosis smoking on patients with lung cancer have led researchers to identify the factors that are associated with continued smoking in these patients. These factors include being of younger age (Cooley et al., 2007), having a relatively low income (Hopenhayn, Christian, Christian, Studts, & Mullet, 2013), having higher levels of depression (Hopenhayn et al., 2013), and living with a family member who smokes (Eng et al., 2014; Hopenhayn et al., 2013). However, other studies have reported nonsignificant relationships between these factors and postdiagnosis smoking (Cooley et al., 2012; Park et al., 2012). Most of the abovementioned studies focused on patients with early-stage lung cancer. Thus, their results may not apply to patients with late-stage lung cancer. In studies that have targeted the population with cancer in general, lifestyle factors such as current alcohol consumption (H. K. Yang et al., 2013) and lack of regular exercise (Fujisawa, Umezawa, Basaki-Tange, Fujimori, & Miyashita, 2014) have been found to be associated with continued smoking after diagnosis. Only one study has examined the role of self-efficacy on continued smoking after lung cancer diagnosis (Cooley et al., 2012). Self-efficacy refers to the strength of individuals' beliefs that they will be able to complete the tasks necessary to reach their goals (Bandura, 1986). This expectation of self-efficacy determines whether individuals will initiate and persist with efforts to reach a goal.

Given the differences in the epidemiology of smoking between Western and Asian countries (Jung, Jeon, & Jee, 2016), the prevalence and risk factors associated with continued smoking in patients with lung cancer may also differ. Information regarding the prevalence of various smoking statuses and related factors is lacking for Asian patients with lung cancer. This study extends previous studies by recruiting patients with newly diagnosed lung cancer at various disease stages and adding self-efficacy and lifestyle variables as potential factors affecting postdiagnosis smoking. The aims of this study were to estimate the prevalence of various smoking statuses among patients with newly diagnosed lung cancer and to identify the differences in characteristics between different smoking status groups within a sample of patients with lung cancer in Taiwan. By identifying those at a high risk for continued smoking, the findings of this study may inform the development of a more effective smoking cessation intervention program for patients with lung cancer in Taiwan.

## Methods

### Study Design and Participants

This study analyzed the baseline data from a longitudinal, observational study. Patients with newly diagnosed lung cancer (*N* = 406) were consecutively recruited from three medical centers in northern Taiwan from May 2014 to March 2016. Patients were invited to participate if they met inclusion criteria, including (a) older than 20 years old, (2) newly diagnosed with lung cancer (i.e., at least 1 month before data collection), (c) alert and able to communicate, and (d) agreed to participate. Patients who were confused/disoriented, cognitively impaired, or diagnosed with a mental disorder were excluded. Data were collected at baseline and once per month for the following 6 months. Only baseline data were used in this study.

### Procedure

The study was approved by the institutional review board of the three study sites (approval numbers: TSGH, 2–103–05-032; VGH, 2014–04-003BC; and CGH, 103-2873B). Eligible patients were approached in outpatient or inpatient units by research assistants, who explained the study purpose and procedure. Baseline data were collected after each patient provided written, informed consent.

### Measures

#### Demographic and lifestyle characteristics

Demographic and lifestyle characteristics were collected using a researcher-developed form. Demographic variables included gender, age, educational level (either “junior high school” or “senior high school”), marital status (either “single/widowed/divorced” or “married/living with a partner”), and income status (either “insufficient” or “sufficient/balanced”). Lifestyle/environmental variables other than smoking included exercise habits, alcohol use, living with a smoker, and secondhand smoke exposure at home.

#### Clinical characteristics

Clinical information (cancer type, cancer stage, functional performance, and comorbidities) were collected by reviewing medical charts. Disease stage was dichotomized as early stage (Stages 0–IIIa) and late stage (Stages IIIb–IV). Functional performance was measured using the Karnofsky Performance Status scale (KPS), which has scores ranging from 0 to 100 with an increment of 10. A KPS of 100 indicates “normal; no complaints/no evidence of disease,” a KPS of 80–90 corresponds to patients being able to carry on normal activities and to work with minor signs or symptoms of the disease, and a KPS below 70 indicates that patients are unable to carry on normal activities or do active work and require some degree of assistance (Schag, Heinrich, & Ganz, 1984). In this study, KPS was classified into < 80 and ≥ 80. Comorbidities were measured using the Charlson comorbidity index (Charlson, Pompei, Ales, & MacKenzie, 1987). Patients with a comorbid condition ≥ 1 were recorded as having a history of comorbid disease.

#### Psychological symptoms

Psychological symptoms were assessed using the 14-item Hospital Anxiety and Depression Scale (HADS), which has two 7-item subscales for anxiety and depression. Each item has four response options that are rated according to a 0–3 scale. The range of total possible scores for each subscale is 0–21, with higher scores indicating more anxiety or depression symptoms (Zigmond & Snaith, 1983). The Chinese-version HADS has shown satisfactory reliability and validity (P. Y. Chen et al., 1999; Y. Yang, Ding, Hu, Zhang, & Sheng, 2014). In the current study, the Cronbach's α was .82 for the anxiety subscale and .73 for the depression subscale.

#### Smoking characteristics

Smoking-related characteristics were age at starting to smoke regularly, average number of cigarettes smoked per day, number of years smoked, and self-efficacy for refusing to smoke. Smoking history was measured as “pack-years,” calculated as the number of years smoked multiplied by the average daily number of packs. Exhaled carbon monoxide (CO), which was used as an objective measure of cigarette consumption, was assessed using a MicroCO meter (Cardinal Health, Chatham, Kent, United Kingdom). The MicroCO meter is a handheld, battery-operated device that measures the concentration of CO on the breath. Participants were instructed to take a deep breath and hold it for 10 seconds and then exhale slowly and fully into a disposable mouthpiece. The exhaled CO concentration is detected by a sensor in the MicroCO meter.

Self-efficacy for refusing to smoke was assessed using the Chinese-version Quitting Self-Efficacy Questionnaire (Cheng & Lee, 2009), which is based on the English-version Smoking Self-Efficacy Questionnaire (Etter, Bergman, Humair, & Perneger, 2000). The Quitting Self-Efficacy Questionnaire assesses the degree of confidence that a respondent has in refusing to smoke during 13 smoking-inducing situations, for example, the item “*When I feel anxious and nervous, I am confident that I can refrain from smoking to ease my anxiety.*” The degree of confidence in each situation is rated on a 5-point scale, where 1 = *not at all confident*, 2 = *30% confident*, 3 = *50% confident*, 4 = *70% confident*, and 5 = *extremely confident*. Total scores range from 13 to 65, with higher scores indicating higher levels of self-efficacy for refusing to smoke. In this study, the Cronbach's α was .97.

#### Smoking status

On the basis of their smoking history, patients were first classified into never-smokers and those ever-smokers. The distinction between never- and ever-smokers was based on the criteria proposed by the Centers for Disease Control and Prevention (Schoenborn & Adams, 2010). Those who reported they never smoked or had smoked < 100 cigarettes in their lifetime were considered as never-smokers. Those who reported that they had smoked > 100 cigarettes in their lifetime were considered as ever-smokers. Ever-smokers were defined as “former smokers” if they had quit smoking more than 1 year before diagnosis. This definition was based on a review finding that quitting smoking for at least 1 year was closely associated with lifelong abstinence (Hughes et al., 2003). Those who had quit smoking for more than 1 month but less than 1 year since diagnosis were classified as “recent quitters.” Those who had quit smoking < 1 month after diagnosis or who still smoked at the time of the interview were defined as “current smokers.”

#### Statistical analysis

Study variables were analyzed using descriptive statistics (percentage, mean, and standard deviation). We first identified factors discriminating between never- and ever-smokers and then identified the risk factors that distinguished current smokers from former smokers and recent quitters. Univariate analyses such as chi-square test, Student's *t* test, and one-way analysis of variance were applied to identify the potential factors that were associated with different smoking statuses. To prevent missing potential influencing factors, a liberal *p* value of < .20 was used to include factors from the univariate analysis (Maldonado & Greenland, 1993) for multivariate analyses with either logistic regression (never- vs. ever-smokers) or multinomial logistic regression (current smokers vs. former smokers or recent quitters). In multivariate analyses, the backward stepwise deletion method rather than the forward method was used to prevent omitting important factors from the final model (Vittinghoff, Glidden, Shiboski, & McCulloch, 2012). Statistical significance was considered when *p* < .05. Data were analyzed using SPSS Version 22 (IBM, Armonk, NY, USA).

## Results

### Sample Characteristics

Among the 406 participants, 191 (47%) had never smoked and 215 (53%) had smoking experience. Among the 215 ever-smokers, 82 (38%) were former smokers who had quit smoking for more than 1 year, 39 (18%) were recent quitters who had stopped smoking within the past 1 year, and 94 (44%) were current smokers who had continued smoking after diagnosis.

The mean age was 64.26 (*SD* = 11.70) years. Over half (57.6%) of the participants were male; 78.8% were married or partnered; most reported an income status of “sufficient,” with 13.1% reporting insufficient income; most (95.6%) were diagnosed with non-small-cell lung cancer; and more than half (61.2%) were in the early stage of their disease. Around half (45.1%) had one or more comorbidities. Most patients (85.5%) had fairly good functional status (KPS ≥ 80). In terms of lifestyle characteristics, 61.1% reported exercising regularly and 18.7% reported drinking alcohol regularly. More than one third (37.9%) of the participants were living with a smoker, but only 30.3% reported being exposed to secondhand smoke at home. Anxiety and depression scores based on the HADS were 4.01 (*SD* = 3.99) and 4.04 (*SD* = 3.72), respectively. Compared with never-smokers, ever-smokers tended to be older and male, to report their income as insufficient, to be diagnosed with small-cell lung cancer, to have a late-stage disease, to have ≥ 1 comorbidity, to have poor functional performance (KPS < 80), to lack exercise habits, to drink alcohol, and to have lower anxiety scores (Table 1).

**TABLE 1.**
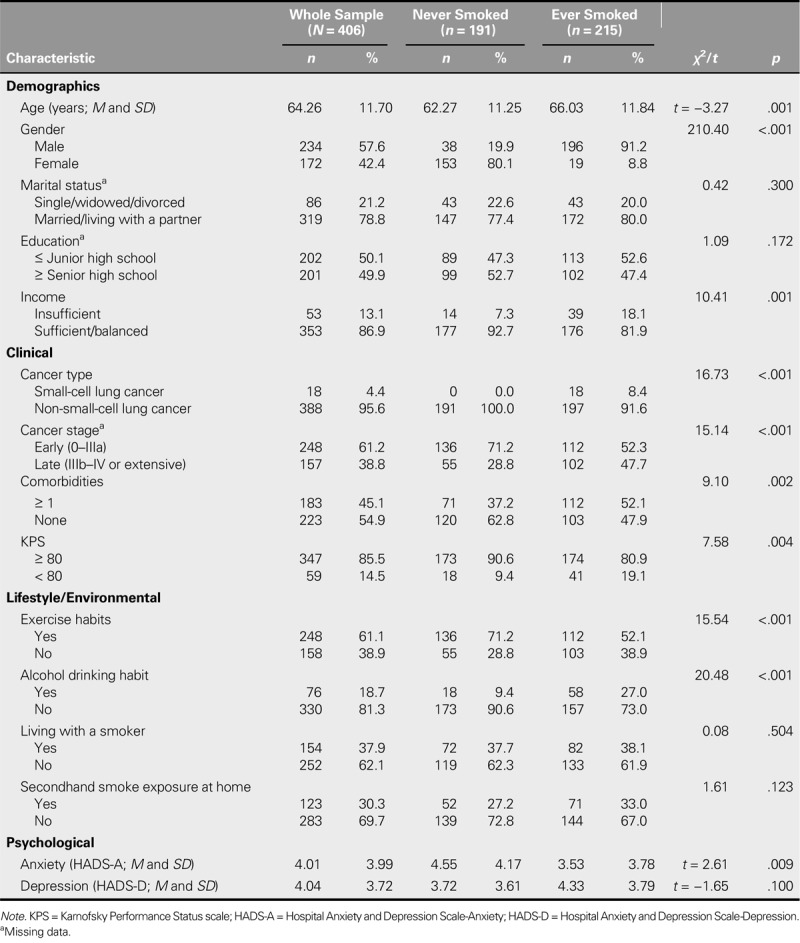
Demographic, Clinical, Lifestyle, and Psychological Characteristics of Never- and Ever-Smoking Patients With Lung Cancer (*N* = 406)

Former smokers were significantly older and had a lower number of total years smoked than recent quitters and current smokers. Current smokers, compared with former smokers and recent quitters, were less likely to be married and to exercise regularly, had a higher mean depression score, had lower self-efficacy for refusing to smoke, and had higher concentrations of exhaled CO (Table 2).

**TABLE 2.**
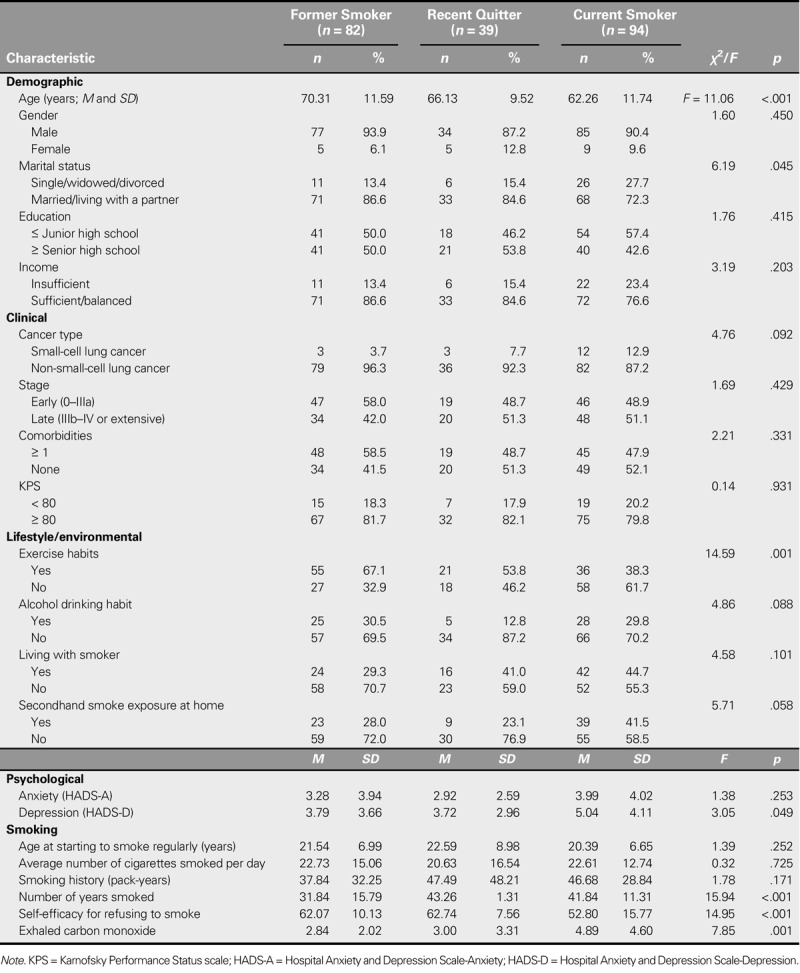
Demographic, Clinical, Lifestyle, and Psychological Characteristics of the Three Categories of Ever-Smoking Patients With Lung Cancer (*N* = 215)

### Factors Discriminating Never-Smokers From Ever-Smokers

Logistic regression revealed that participants were more likely to be an ever-smoker if they were male (*OR* = 53.98, 95% CI [27.32, 106.66]), had at least one comorbidity (*OR* = 2.49, 95% CI [1.35, 4.60]), had KPS < 80 (*OR* = 2.93, 95% CI [1.22, 7.03]), had no exercise habits (*OR* = 2.01, 95% CI [1.09, 3.69]), or were exposed to secondhand smoke (*OR* = 2.21, 95% CI [1.12, 4.36]; Table 3).

**TABLE 3.**
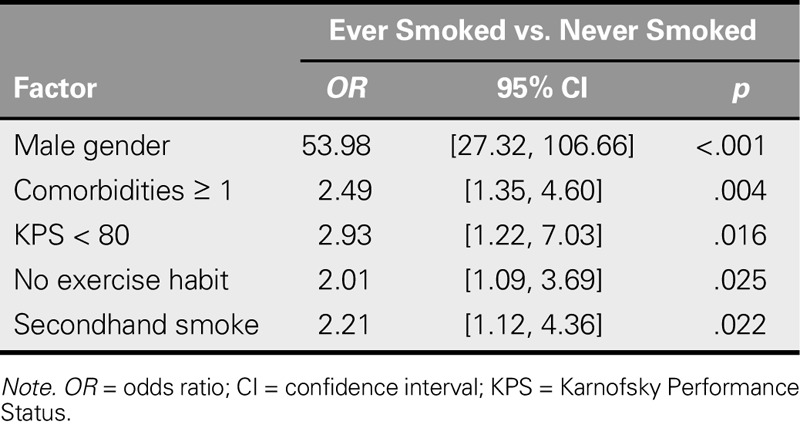
Factors Distinguishing Ever-Smokers From Never-Smokers (*N* = 406)

### Factors Associated With Being a Current Smoker After Lung Cancer Diagnosis

Multinomial logistic regression revealed that participants were more likely to be a current smoker than a former smoker if they were younger (*OR* = 1.05, 95% CI [1.02, 1.09]), had no exercise habits (*OR* = 2.74, 95% CI [1.37, 5.47]), lived with a smoker (*OR* = 2.48, 95% CI [1.22, 5.04]), or had lower self-efficacy for refusing to smoke (*OR* = 0.95, 95% CI [0.92, 0.98]). However, only self-efficacy for refusing to smoke differentiated current smokers from recent quitters. Current smokers were more likely than recent quitters to have lower self-efficacy for refusing to smoke (Table 4).

**TABLE 4.**
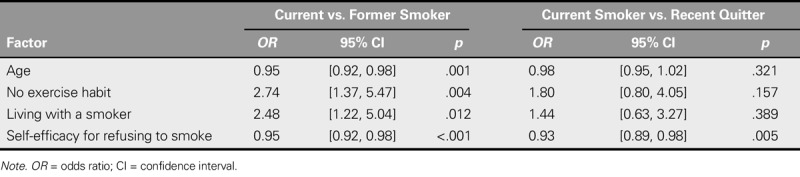
Factors Distinguishing Current Smokers From Former Smokers and Recent Quitters (*N* = 215)

## Discussion

More than half of the present sample of patients with lung cancer were never-smokers. Of the ever-smokers, more than 40% continued smoking after diagnosis. Compared with former smokers, those who continued to smoke after diagnosis were more likely to be younger, be living with a smoker, not exercise regularly, and have lower self-efficacy for refusing to smoke. However, the only factor that differentiated current smokers from recent quitters was self-efficacy for refusing to smoke, with current smokers having significantly lower self-efficacy.

The high prevalence of never-smokers found in this study is consistent with previous reviews that indicate that lung cancer is prevalent among Asians who have never smoked (Couraud et al., 2012; Yano et al., 2008). The percentage of female never-smokers in this study was high (*n* = 153, 80.1%). As the ratio of female-to-male smokers in Taiwan is roughly 1:7 (Health Promotion Administration, Ministry of Health and Welfare, Executive Yuan, Taiwan, ROC, 2016a), this may explain why the prevalence of never-smokers in Asian countries is high. Environmental and genetic factors have been suggested for the high prevalence of lung cancer among Asian female nonsmokers (Ha et al., 2015; Samet et al., 2009). The prevalence in this study of patients who continued smoking after lung cancer diagnosis (43.7%) is similar to that (48.7%) reported for U.S. patients with lung cancer (Baser et al., 2006).

The finding that younger age is associated with continued smoking after diagnosis is similar to a report that younger Taiwanese smokers at a smoking cessation clinic were less likely to abstain from smoking because they participated in more activities where smoking is socially encouraged and had a higher likelihood of being surrounded by smokers (Cheng & Lee, 2009). However, the finding in this study on age contrasts with previous reports that found no association between age and continued smoking in patients with lung cancer (Cooley et al., 2012; Hopenhayn et al., 2013). Loss of physical function with age may explain these findings. Decline of physical function was reported to be the major predictor of smoking cessation among 50- to 66-year-old Taiwanese smokers without lung cancer (Tsai, Lin, & Tsai, 2012). Another possible explanation is that younger smokers tend to have lower risk perceptions of getting cancer than older smokers (Peretti-Watel et al., 2007), and smokers who perceive that smoking is a significant hazard to health are more inclined to abstain from or quit smoking (Jiang, Elton-Marshall, Fong, & Li, 2010; Schnoll, Subramanian, Martinez, & Engstrom, 2011).

The finding in this study that participants who did not exercise regularly were more likely to continue smoking is consistent with previous reports that short bouts of physical activity or exercise reduce smokers' cigarette cravings (Fong, De Jesus, Bray, & Prapavessis, 2014; Haasova et al., 2013). Therefore, regular physical activity may be added to smoking cessation programs to enhance program effectiveness.

Moreover, the finding that living with smokers increased the probability of continuing to smoke is consistent with previous reports (Hopenhayn et al., 2013; Schnoll et al., 2002). Exposure to secondhand smoke has been shown to activate nicotine receptors in the brain, which may increase smoking desire and the risk of nicotine dependence, thus promoting continued smoking (Brody et al., 2011; Okoli, Browning, Rayens, & Hahn, 2008). Observing other smokers' behaviors may also stimulate ex-smokers to resume their smoking behavior. This possibility is supported by reviews that have shown that smokeless workplaces encourage smokers to quit smoking or reduce their amount of smoking (Cahill & Lancaster, 2014). Therefore, reducing patients' exposure to secondhand smoke at home and in the workplace may help them quit smoking.

Another finding from this study was that continued smoking after a lung cancer diagnosis was significantly associated with low self-efficacy for refusing to smoke, which is also similar to the results of previous studies (Cooley et al., 2012; Schnoll et al., 2011). Patients with lung cancer who have higher self-efficacy for refusing to smoke are more likely than those with lower self-efficacy to take action to quit smoking and to continue to abstain from smoking. The outcome expectation that quitting will benefit disease prognosis may also enhance self-efficacy to reduce or quit smoking. Because self-efficacy for refusing to smoke and smoking status were assessed simultaneously, this study cannot examine the possible causal relationship between self-efficacy and smoking status. Indeed, a 54-study meta-analysis found that the relationship between self-efficacy and successful smoking cessation was mitigated by the duration between the assessments of self-efficacy and outcome (Gwaltney, Metrik, Kahler, & Shiffman, 2009). When smoking status was controlled at the time of self-efficacy assessment, the relationship between self-efficacy and future smoking behavior was found to be much weaker (Gwaltney et al., 2009). The potential of using self-efficacy as a predictor for refusing to smoke in patients with lung cancer and the factors that affect self-efficacy should be investigated further.

### Limitations

This study had several limitations. First, participants were not randomly sampled, which may limit the generalizability of the results. However, this limitation may be offset by our sampling of multiple medical centers. Second, this study used a cross-sectional design, precluding inferences about causal relationships between the factors and smoking status. Third, participant smoking status was determined using self-reported data, which may be biased by social desirability. However, this limitation is counterbalanced by the results showing the amounts of CO exhaled by participants who continued to smoke to be higher than the amounts exhaled by either recent quitters or former smokers.

### Conclusions

Nearly half of the patients with newly diagnosed lung cancer who were ever-smokers were active smokers at the time of diagnosis. The risk factors that were identified for patients with lung cancer who continued to smoke after diagnosis included lack of exercise habits, living with a smoker, and low self-efficacy for refusing to smoke. We suggest promoting regular exercise and enhanced self-efficacy in future smoking cessation interventions for patients with newly diagnosed lung cancer to increase the rate of smoking cessation in this vulnerable group.
